# Understanding Influencing Factors of Travel Mode Choice in Urban-Suburban Travel: A Case Study in Shanghai

**DOI:** 10.1007/s40864-023-00190-5

**Published:** 2023-03-26

**Authors:** Jiankun Le, Jing Teng

**Affiliations:** 1 Urban Mobility Institute, Tongji University, 4800 Caoan Highway, Shanghai, People’s Republic of China; 2 Key Laboratory of Road and Traffic Engineering of the Ministry of Education, College of Transportation Engineering, Tongji University, 4800 Caoan Highway, Shanghai, People’s Republic of China; 3 Shanghai Collaborative Innovation Research Center for Multi-network and Multi-modal Rail Transit, Tongji University, Shanghai, 201804 People’s Republic of China

**Keywords:** Travel mode choice, Urban-suburban transportation corridors, Discrete choice models, Influencing factors, Machine learning algorithm, RP and SP survey

## Abstract

After the rapid expansion of the subway system over the past two decades, some cities are preparing to build more suburban railways. The emergence of suburban railways is bound to change the choice of suburban passenger transportation. This paper studies the factors that affect the choice of travel mode at the construction stage of suburban railways, aiming to design a more rational suburban railway network and urban public transport service system. Taking Shanghai as an example, this study first surveyed revealed preference (RP) and stated preference (SP) among urban-suburban travelers. Then, we used discrete choice models (DCM) and machine learning algorithms to build a travel mode choice model based on data collection and analysis. Furthermore, the importance of each factor was analyzed, and the effects were predicted under several traffic demand management schemes. Finally, this study proposed some strategies for increasing the share of public transport. On the one hand, it is suggested that Shanghai should continue to develop suburban railways and maintain low pricing of public transport services. Considering the construction and operation costs, the government needs to provide certain subsidies to stabilize prices. On the other hand, as passengers are very sensitive to the “last mile” trips in their suburban railway travel, transport planners should strengthen the connection from and to the suburban railway stations by developing services such as shared bikes and shuttle buses. In addition, the results indicated that some traffic demand management measures can also contribute to a larger share of public transport.

**Supplementary Information:**

The online version contains supplementary material available at 10.1007/s40864-023-00190-5.

## Introduction

In the big cities of Western Europe and Japan, the well-developed multilevel urban public transport network has become one of the urban residents' main daily travel choices. In contrast, the fast and intensive construction of subway networks is still on the way in China and other developing countries. These cities are likely to enter a suburban railway construction boom in the coming decades [1]. Thus, it is necessary to study how the newly opened suburban railway will change the urban-suburban travel pattern of the city.

This paper aims to study the choice behavior of passenger travel mode in the suburban passenger corridor under the background of the suburban railway construction boom in China's big cities. This paper focuses on the influencing factors of the choice of suburban passenger travel modes. Taking Shanghai as an example, from the whole process of urban-suburban travel, the discrete choice model and machine learning method are used to construct various travel mode choice models, which are further evaluated and compared. Finally, we propose some strategies to enhance the competitiveness of urban-suburban public transport services based on the analysis.

The innovation of this study is to use both traditional and improved logit models and two machine learning methods. This study combines the results of various models to obtain more findings than those from the traditional discrete choice model. The results of multiple models can corroborate or complement each other.

The significance of this study is to investigate the travel behavior on urban-suburban passenger transportation corridors, taking Shanghai as a case study. Suggestions to enhance sharing rates of public transit are proposed. The constructed models can provide quantitative support for the design of various aspects of speed, fares, and connecting traffic in the planning and operation of suburban railways.

The rest of the paper is organized as follows. Section 2 reviews related literature. Section 3 demonstrates the methods adopted in the study. Section 4 presents the survey design with variables and scenarios. In Section 5, using discrete choice models and machine learning algorithms, the modeling results are introduced, discussed, and summarized. Section 6 predicts the effects of various traffic demand management schemes based on the models from the two methods and gives suggestions to enhance the competitiveness of urban-suburban public transport; finally, Section 7 concludes the study.

## Literature Review

### Urban-Suburban Travel Mode Choice

Urban-suburban travel refers to moving between the main sections of a city (urban area/main area) and the suburbs of the city (new town). Urban-suburban travel usually covers longer distance and time, and possibly has more travel links than those that start and end in central or suburban areas. The differentiation between urban and suburban areas in comprehensive transportation construction also complicates the travel mode selection process. Travelers need to make decisions based on a variety of factors.

At present, there are few studies on urban-suburban travel choices, especially considering the accessibility of public transport services and the impact of new suburban railways on mode choice.

Espino et al. [2] used the RP/SP (revealed preference/stated preference) method to investigate travelers on suburban railway channels and analyzed the impact of several policies on the share rate of public transport. Monson and Gonzales [3] predicted the first year of passenger flow of the N-III passenger corridor in Madrid and found that the sharing rate of various transportation modes on the corridor would change significantly.

In the research on the suburban railway system in China, there needs to be more research on the travel choice behavior of suburban railways. Tiantian and Yaodong [4] studied the convenience of public transportation for suburban commuters in Beijing in 2021. Using the structural equation model, she found different perceptions of convenience for commuters with different characteristics and put forward suggestions for promoting suburban public transportation.

In some developing countries or regions where the composition of public transit systems differs from that of developed countries, travel mode choices for suburban trips may also differ. Only a few studies on suburban travel behavior are currently being conducted in developing regions. Danapour et al. [5] studied the impact of high-speed rail on travel demand for Tehran–Isfahan routes and used the SP survey method to obtain passengers' attitudes toward changing to high-speed rail travel. Dahlan and Fraszczyk [6] conducted a pre-launch study of the Jakarta Mass Rapid Transit (MRT) system through a survey in Jakarta, Indonesia, and three surrounding areas, and found that most of the respondents declared a willingness to a shift to MRT. Fraszczyk et al. [7] studied people’s willingness to shift to metro through a survey targeting a group of potential metro users located close to a planned metro line in Salaya, 20 km from the center of Bangkok, Thailand. Tiantian and Yaodong [4] studied passenger convenience during the transfer process from a more micro perspective by using questionnaires for passengers in the suburbs of Beijing and a structural equation model. The empirical results show that passengers' perception of the convenience of transfer in the suburban commute is characterized by comfort, safety, and quickness, with comfort coming first, then safety, and quickness last. The subjects of these studies may be close to urban-suburban travel, but they focused more on metro and less on the suburban railway.

### Disaggregate Travel Survey Methods

The revealed preference (RP) and stated preference (SP) surveys are the more commonly used methods in travel behavior research. In recent years, researchers have used these methods to study various types of travel behavior.

Using the above RP or SP survey method, Cherchi analyzed the future travel-sharing rate of a railway in Italy that was about to be opened. Espino et al. [2] studied the travel mode choice behavior in two main urban-suburban travel corridors in Gran Canaria, Spain, and found that punishing car travel was more effective in stimulating the public's choice of public transport than improving the service of public transport. Ali Aden et al. [8] aimed to investigate the intentions and preferences of travelers toward car-sharing services in Djibouti, Africa, and the data were collected through an online stated preference (SP) survey. Shamshiripour et al. [9] designed an SP-RP survey implemented in Chicago to investigate how and to what extent people's mobility styles and habitual travel behaviors had changed during the COVID-19 pandemic.

### Behavioral Theory of Travel Mode Choice

#### Discrete Choice Model (DCM)

The discrete choice model (DCM) has been widely used in the study of travel choice. To date, discrete choice model theory has generally been classified into multinomial logit model (MNL), generalized extreme value model (GEV), mixed logit model, and multinomial probit model. Many transportation scholars have studied and applied DCM combing their own research needs, such as the multiple discrete continuous extremum model (MDCEV) [10], structural equation-based logit model (SEM-Logit) [11], and integrated choice and latent variable model (ICLV) [12].

#### Machine Learning Methods

In addition to the DCM, in recent years, many scholars have begun to use other models to analyze the travel choice problem, among which the supervised learning model in the machine learning type is more common.

Among them, the support vector machine (SVM) algorithm has developed rapidly since 1995 and has given rise to a series of extended algorithms [13], and has been widely used in data analysis and mining modeling of intelligent transportation systems in recent years. Cheng et al. [14] proposed a travel mode prediction method based on SVM and analyzed the travel mode choices of low-income commuters in Fushun. In order to verify the hypothesis that the household head's travel behavior will impact the travel frequency of his family members, Yang et al. [15] applied the MNL model and SVM model, respectively, when taking the travel data of Nanjing residents as a case. They found that the SVM performed slightly better than the overall average accuracy by comparing the prediction accuracy. Qian et al. [16] proposed a new SVM classification method, aiming to classify the travel mode selection data more accurately so that the model could predict the travel mode more accurately.

The random forest algorithm [17] is a combinatorial classification method proposed by Leo Breiman in 2001. Due to its ease of use and high prediction accuracy, it has been gradually applied in transportation science. Cheng et al. [18] proposed using the random forest algorithm to predict travel behavior and conducted a case study with household travel data in Nanjing.

## Research Gap

In the early stage of suburban railway development in developing countries, there were few published results related to the travel choice behavior of suburban railway or urban-suburban passenger transport corridors.

The purpose of this work is to conduct a case study of Shanghai's urban-suburban passenger transport corridor on travel choice behavior. The study uses the classical discrete choice model and machine learning methods to investigate the characteristics of urban-suburban travel mode choice. Then we put forward related travel demand management policies based on these characteristics and predict the effect by different models.

In contrast to the previous literature, on the one hand, this study uses Shanghai (a city in a developing country where urban railways are being built) as a case study, while the previous literature has paid less attention to urban railways in developing regions, and has also studied less about suburban railways or urban-suburban corridors. On the other hand, this study experimented with machine learning methods and the traditional discrete choice models in its methodology. Additional findings are obtained by evaluating the results of the two methods.

## Method

### Discrete Choice Model (DCM)

The theoretical basis of the DCM is that people choose the option with the greatest “utility” when they make a choice (the random utility theory). In the case of transportation, the sample option of “utility” of the same option differs for different travelers. The characteristics of the level of service, the attributes of the traveler, the purpose, and the cost of the trip all impact the traveler's perception of the “utility” of the option.

Random Utility Theory also assumes that “utility” is not a fixed value, but a random variable with a nonrandom and a random component, as Equation (Eq. 1) below.

1 $$U = V + \varepsilon$$

where *U* represents the utility of a solution for a traveler, *V* represents the fixed part of it and $\varepsilon$ represents the unfixed part.

As shown in Eq. (2) below, according to utility maximization theory, the probability that the traveler will choose one option $i$ is

2 $$P_{i} = \Pr {\text{ob}} \left( {U_{i} > U_{j} ;i,\;j \in A} \right) = \Pr {\text{ob }}\left( {V_{i} + \varepsilon_{i} > V_{j} + \varepsilon_{j} ;\; i,\;j \in A} \right)$$

where A is the set of all available options. $0\le {P}_{i}\le 1$, $\sum_{i\in A}{P}_{i}$.

The observable component ${V}_{i}$ is generally considered to be the sum of the product of the variable ${X}_{k}$, representing the influencing factor, and its weight parameter ${\alpha }_{k}$, as shown in Eq. 3, is

3 $$V_{i} = \sum {\alpha_{k} \cdot X_{k} }$$

The basic DCM builds mathematical models based on the above assumptions, using mathematical methods to identify the variables ${X}_{k}$ that significantly impact travel utility and solve for their impact parameters ${\alpha }_{k}$.

In general, the main models for discrete selection are the MNL, the GEV, the mixed logit model, and the multinomial probit model. Since 2010, many scholars have developed and applied complex models for different data forms and problem forms based on these common basic models.

Based on the frequency of use of each type of model in the study, the match with the sample data collected so far, and the effectiveness of the parameter estimation demonstration, the following types of DCM are selected for parameter estimation and testing in this study to conduct comparisons between modeling results.

1. The basic multinomial logit model

The MNL is a simple discrete choice model in which random utilities are set to follow independent extreme value distributions. The choice probability function is $P ={ e}^{\beta x}/\Sigma { e}^{\beta x}$, where the explanatory variable $x$ can be either the individual socioeconomic characteristics of the chooser or the attributes of options.

The MNL model is the basis of the discrete choice model system, and several types of logit models have been improved to fix some of the drawbacks of the MNL model. In practical research, MNL models are also the most widely used due to their ease of use and low error rate.

(2) The willingness-to-pay MNL model

The difference between the willingness-to-pay MNL model and the basic MNL model is that the levels of travel modes (such as time and comfort) can be multiplied by a parameter and added to the cost of travel, thus considering the cost of time and the perceived cost in the model.

In this study, the willingness-to-pay MNL model can provide a clearer, quantitative response to passenger perceptions of factors such as time and comfort under different travel modes, and support the proposal of relevant travel management strategies.

(3) The perceived-time MNL model

The perceived-time MNL model differs from the basic MNL model in that it assumes that crowding, possible delays, and the distance from the location to the station will affect the passenger's perception of time. For example, passengers may perceive a longer journey time in a very crowded and uncomfortable carriage.

The perceived-time MNL model can multiply a number of travel mode attributes by their coefficients, add or multiply them with time, and subsequently obtain the total perceived time of the traveler. This model can be used to analyze the effect of improvement methods such as comfort enhancement on reducing perceived time.

(4) The nested logit model

The MNL models have the inherent flaw of the assumption of IIA (independence of irrelevant options). When the IIA hypothesis is not tested, MNL cannot be used for modeling. The initial focus of academic research in DCM is to solve the IIA property, which leads to the development of the GEV. The GEV model is not a specific model but a generic term, with the most widely used model in practice being the nested logit model (NL).

The NL model places options that share specific characteristics in the same category. The questionnaire in this study classifies urban public transport as “rail transit” and “ground bus,” but there are many similarities between the two in terms of various factors, and several connection modes are common. Therefore, in the NL model, the two can be classified in one category.

### Support Vector Machine (SVM)

Support vector machine (SVM) is a supervised learning algorithm for data classification. Its principle can be briefly summarized as follows. The training samples are separated by representing the points in the feature space with different coordinates, followed by finding the best plane (hyperplane) in the feature space that separates all the sample points. The advantage of SVM is that it can learn from small samples, emphasizes key vectors without being too sensitive to outliers, and has a strong generalization capability.

This study divides the dataset into a training set and a test set, allowing the SVM to learn prediction rules for travel mode choice from the training set data. Then, the model obtained from the training predicts the final chosen travel mode from the samples in the test set.

### Random Forest

Random forest is also a supervised learning algorithm. The principle can be explained as follows. There is a forest made up of a group of decision trees. First, features are randomly selected for the trees, and the importance of each feature in each tree is tested. Then, all the decision trees are iterated toward the more important features. Finally, the prediction of the forest is obtained based on the results of all the decision trees. A significant advantage of this method is that it can be used for both classification and regression problems, and random forests can also measure the importance of each feature and give a ranking.

## Research Design

### Research Framework

**Fig. 1:**
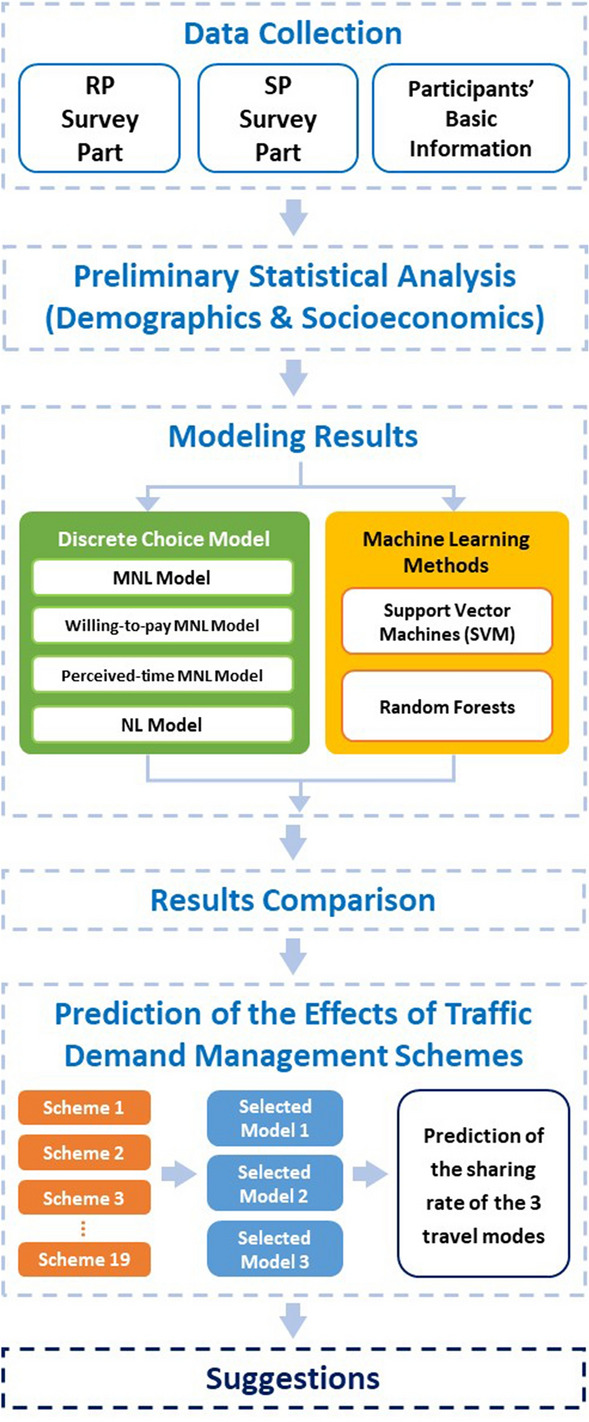
Research framework

As shown in Fig. 1, this paper proposes a research framework. The framework begins with a three-part data collection followed by preliminary statistical analysis (including demographic and socioeconomic analysis). Then, DCM and machine learning methods are used in the modeling part, and a comparison of the results of the two models and some findings are presented. In the last part of the framework, the three best-performing models are selected to predict the change in travel mode choice under 19 different policy schemes. Finally, we summarize the multi-part research and put forward some suggestions to enhance the competitiveness of public transit.

### Study Area: Public Transport and Suburban Railways in Shanghai

Shanghai, a megacity with a permanent population of more than 20 million, already has a metro system with 20 lines and an overground bus system with more than 1000 lines.

Compared with the metro and bus, Shanghai suburban railway planning and construction started later. Shanghai currently operated only one suburban passenger railway, Jinshan Railway. In 2016, Shanghai's suburban railway entered a period of rapid development. Nine suburban railways were under planning and construction, such as the Jiamin Line and the Airport Link Line [19]. Shanghai will have a suburban railway network of more than 1000 km by 2035.

**Fig. 2:**
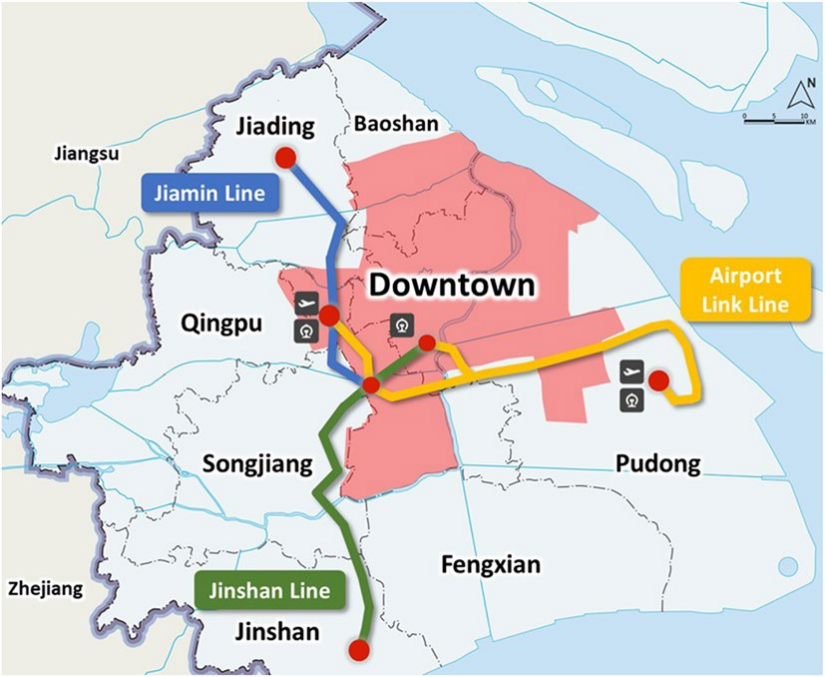
Shanghai's downtown and suburban areas, and the directions of suburban railways

Figure 2, referring to the Shanghai Master Plan 2017–2035 [20], shows the downtown in red and the suburbs in light blue. The map shows the alignment of the three urban railway lines already in operation or due to be completed by 2025, namely the Jinshan Line, Airport Link Line, and Jiamin Line. Each of the lines straddles the urban and suburban areas to support the needs of the public for urban-suburban travel.

In our study, a trip from the downtown to the suburbs (or from the suburbs to the downtown) is considered an urban-suburban trip. Given that this division is unclear to all participants, the questionnaire may use administrative divisions to distinguish between urban and suburban areas.

### RP and SP Survey Questionnaire Design

#### The Structure of the Questionnaire

This study uses quantitative methods where questionnaires serve as data collection tools to conduct data acquisition for suburban travel choice behavior, aiming to study the influence of multiple factors such as traveler attributes, travel attributes, travel mode attributes, and so on. The formal questionnaire design includes three parts: the socioeconomic attributes of travelers, the RP survey of suburban travel choice, and the SP survey of suburban travel choice.

The first part of the questionnaire investigates the socioeconomic attributes of the respondents, asking about seven attributes such as gender, age, occupation, average monthly income of family members, living area, the distance from his or her residence, and the nearest subway station (*D*_RS_), and family car usage.

The second part of the questionnaire is the RP survey. The questionnaire inquiries about the actual suburban trips that occur most frequently, including the travel purpose of this trip, the area of the destination, the distance between the destination and the nearest subway station (*D*_DS_), the one-way travel cost, the one-way travel distance, and the way to reach the public transport station. If the respondents could not give accurate answers to some questions, such as travel cost and distance in the questionnaire, they were asked to make simple estimates.

Attributes of the first and second parts of the survey are defined in Table 1.

**Table 1:** Attributes of the first and second parts of the survey

Attribute type	Attribute name	Description
Personal attribute	Gender	Male = 0, Female = 1
	Age	Under 18 = 1, 18~30 = 2, 31~40 = 3, 41~50 = 4, 51~60 =5, Over 60 = 6
	Per capita monthly household income (Income)	0~5000 yuan = 1, 5001~10,000 yuan = 2, 10,001~20,000 yuan = 3, >20000 yuan = 4
	Work	Non-commuter = 0, Daily commuter =1
	Family car ownership (CarOwn)	Have no car = 0, Own a car = 1
Travel attribute	Travel purpose	Non-commuting = 0, Commuting = 1
Travel mode attribute	Travel time	
	Cost	
	Distance from the respondent’s residence to the nearest station (*D*_RS_)	
	Distance from the respondent’s destination to the nearest station (*D*_DS_)	
	Crowded degree	
	Possible delay	
	Number of transfers	
	Shared bikes available ${(AV}_{Bicycle})$	Available=1, Unavailable=0
	Shuttle buses available $({AV}_{Shuttle})$	Available=1, Unavailable=0
	Park and Ride (*P*+*R*) available $({AV}_{Parking})$	Available=1, Unavailable=0

The third part of the questionnaire is the SP survey. The questionnaire gives a hypothetical scenario. Each scenario has three travel modes (rail transit, car, bus), in which rail transit includes subway, suburban railway and so on, and the car includes taking taxis and self-driving. Each mode's attributes (time in transit, one-way cost, possible delays, *D*_RS_, *D*_DS_, and so on) are assumed. Respondents were asked to choose one of the three travel modes in each scenario.

Twenty-four scenarios were generated using an orthogonal design. Six of them were randomly selected for each respondent to answer. Respondents were asked to answer the following five questions with three options (rail transit, car, bus) for each scenario.

1. In general, what is your chosen travel mode?
2. If a shuttle bus is opened between the place of residence and the station with greater frequency and appropriate time, the one-way cost will increase by up to 3 yuan. What would be your travel mode in this situation?
3. If the shared bicycles are placed near the residence and the station, the number is large, easy to park, and the travel cost is almost unchanged. What would be your travel mode in this situation?
4. If there is a special public parking lot (*P*+*R* parking lot) next to the station with ample parking space available, and you can directly transfer to the subway after parking, the parking fee is 10 yuan/day. What would be your travel mode?
5. If there is a railway from the city to the suburbs, when choosing “subway or train,” the travel time can be shortened to 60 min, and the single trip cost becomes 15 yuan. What would be your travel mode?

#### The Attributes and Levels Design of the SP Survey

In addition to the common attributes of travel purpose, transit time, and travel cost, the attributes selected for the SP questionnaire include distance, congestion, number of interchanges, and possible delays. *D*_RS_ and *D*_DS_ are considered to investigate the relationship between the convenience of the passenger reaching the station and his or her choice of travel mode.

For the level design of each attribute, this study obtained the average level and range of variation through the official website of Shentong Metro, Jinshan Railway, Gaode Map software, the Didi TNC application, and references. For example, the level of travel time is limited to between 40 min and 120 min because this is the commuting time of the vast majority of Shanghai residents mentioned in *The 2022 Commuting Monitoring Report of Major Cities in China* [21]. The cost of public transport, on the other hand, is generally in the range of 2–15 yuan, which is determined by considering the pricing of Shanghai's metro, Jinshan Line, ground buses, and company buses.

Indicators that are difficult for respondents to visualize are described in a verbalized way (e.g., crowdedness, when it is 3, 6, and 10 people/m^2^ is described in the questionnaire as “have seats, not crowded,” “no seats, crowded,” and “no seats, very crowded,” respectively [22–24]).

The attributes and levels of the SP questionnaire are designed as follows. Table 2 also explains what the different levels represent or where they come from.

**Table 2:** The attributes and levels of the SP questionnaire

Attribute	Level	Meaning
Travel purpose	1	Commuter travel
	0	Non-commuter travel
*Rail transit*		
Travel time	40 min	Suburban railway direct train
	60 min	Suburban railway+subway
	90 min	Subway
	120 min	Subway
Cost	5 Yuan	One-way subway discounted price
	10 Yuan	One-way subway price
	15 Yuan	One-way suburban railway price
Distance from the respondent’s residence to the nearest station (D_RS_)	1 km	In walking distance
	3 km	Probably need connecting traffic
	5 km	Need connecting traffic
Distance from the respondent’s destination to the nearest station (D_DS_)	1 km	Within walking distance
	3 km	Probably need connecting traffic
Possible delay	5 min	High punctuality of Shanghai metro
Crowded degree	3 people/m^2^	Have seats and not crowded
	6 people/m^2^	No seats and crowded
	10 people/m^2^	No seats and very crowded
Number of transfers	0	Direct train
	1, 2	Not a direct train
*Car*		
Travel time	40 min, 60 min, 90 min	
Cost	30 Yuan	Driving/carpooling
	50 Yuan	Driving/taxi/car-hailing
	80 Yuan	Taxi/car-hailing
Possible delay	15 min, 30 min	
*Ground bus*		
Travel time	40 min	Direct bus
	60 min	Main station express bus/bus rapid transit (BRT)
	90 min	Bus
	120 min	Bus
Cost	2 Yuan	Bus
	5 Yuan	Main station express bus/BRT
	10 Yuan	Chartered bus/company bus
*D*_RS_	1 km	Shanghai's ground bus system has a denser stop network than metro
*D*_DS_	0.5 km	
Possible delay	15 min, 30 min	
Crowded degree	3 people/m^2^	Have seats, not crowded
	6 people/m^2^	No seats, crowded
	10 people/m^2^	No seats, very crowded
Number of transfers	0	Direct bus
	1, 2	Not a direct bus

#### The Scenario Designs

In designing the scenarios, the orthogonal design function of the statistical analysis software JMP Pro 13 is used to generate 24 choice scenarios, rejecting some combinations of factor levels that are too unattractive (e.g., highest cost but slowest speed). Due to interview length constraints and to improve the validity of the respondents' answers, each respondent answers five questions in six of the 24 scenarios randomly.

### Data Collection and Sample Size Estimation

The questionnaire survey was conducted on residents living in the suburbs of Shanghai for a long time. We informed respondents at the beginning of the questionnaire that the travel behavior surveyed was for a period when COVID-19 outbreaks had not yet occurred (no large-scale COVID-19 outbreaks in Shanghai from March 2020 to March 2022).

Affected by the COVID-19 epidemic in Shanghai in 2022, this questionnaire survey was conducted in the form of an online survey in April 2022, and a professional survey company was commissioned to conduct the survey. After deleting some invalid results, A total of 575 valid questionnaires were collected. The following is a preliminary analysis of the survey data. After processing the data, a total of 15,540 valid travel mode choice data were generated.

Based on the following inequality Eq. 4 proposed by Orme [25] and Johnson and Orme [26] applicable to simple random sampling, this inequality can be used to determine sample size in SP surveys, taking into account crossover effects between factors.

4 $$N \ge 500 \cdot \frac{{L_{MAX} }}{J \cdot S}$$

where ${L}_{MAX}$ is the maximum value of the product of the number of levels of the two attributes, $J$ is the number of options, and $S$ is the number of choice tasks faced by each respondent.

The maximum value of the number of levels for a single attribute in the questionnaire is 4 and consists of two such attributes, so this survey has ${L}_{MAX}=16$. The respondent has to choose between three modes of travel, so $J=3$; each respondent answers the questions in six scenarios, so $S=6$. Therefore, the minimum value of the sample size is $500\cdot \frac{16}{3\times 6}\approx 445$. This survey collected 575 valid questionnaires, which met this requirement in terms of quantity.

## Analysis of Results

### Statistical Preliminary Analysis

#### Participants’ Demographic and Socioeconomic Characteristics

**Fig. 3:**
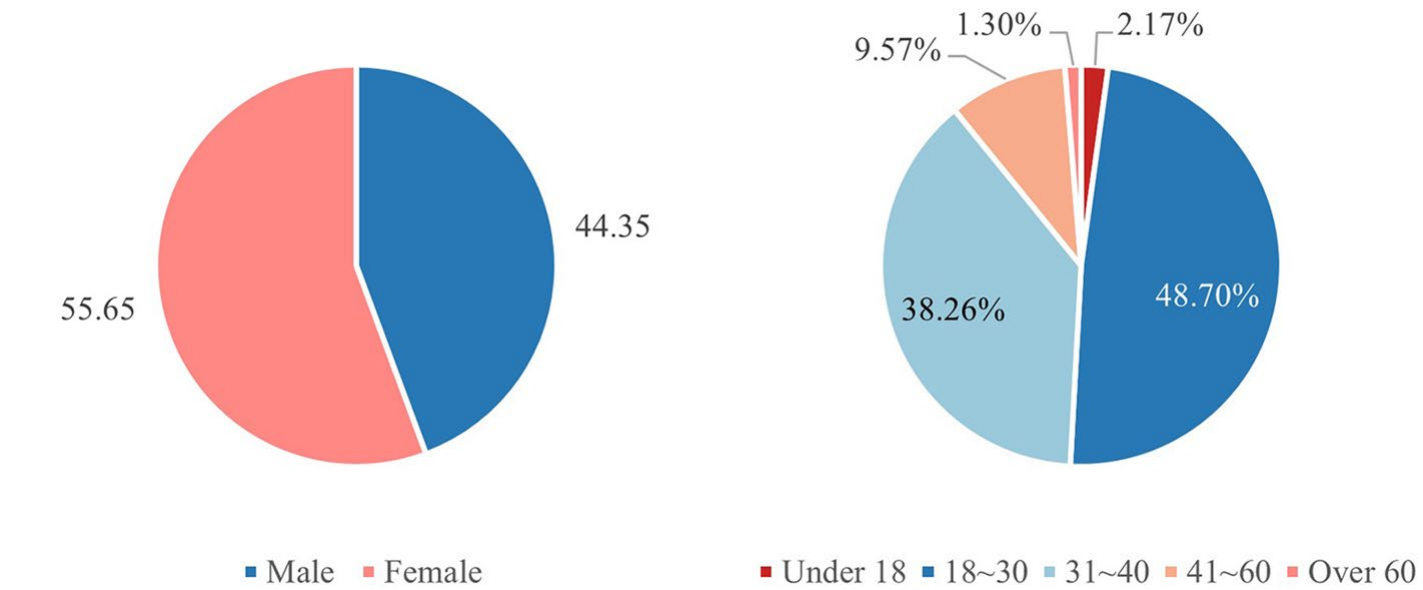
Gender (left) and age (right) of respondents for sample collection

The basic personal characteristics of the respondents in this survey were analyzed. As shown in Fig. 3, from the perspective of gender, male respondents account for 44.35%, female respondents account for 55.65%, and the overall gender distribution is relatively balanced. From the perspective of age, 2.17% are under 18 years old. Respondents aged 18–30 and 31–40 years account for 48.7% and 38.26%, respectively.

Participants are relatively evenly split in terms of gender, and the majority of respondents are between the ages of 18 and 59, which is the most active age group in travel behavior. It may also be related to the limitation that the elderly have fewer travel times, and many online surveys are difficult to cover the elderly.

Monthly per capita household income is a better indicator of overall affordability than personal income, so it is chosen as the income item (referred to as “income” in subsequent research and analysis) for the survey. The results are more evenly distributed across the income bands.

Regarding occupation, general employees, enterprise managers, and students account for a large proportion, reaching 39.13%, 21.3%, and 19.57%, respectively. Public data from the Shanghai government shows the average monthly salary in Shanghai is around 12,000 yuan, and the median is around 6400 yuan, which aligns with the survey results.

**Fig. 4:**
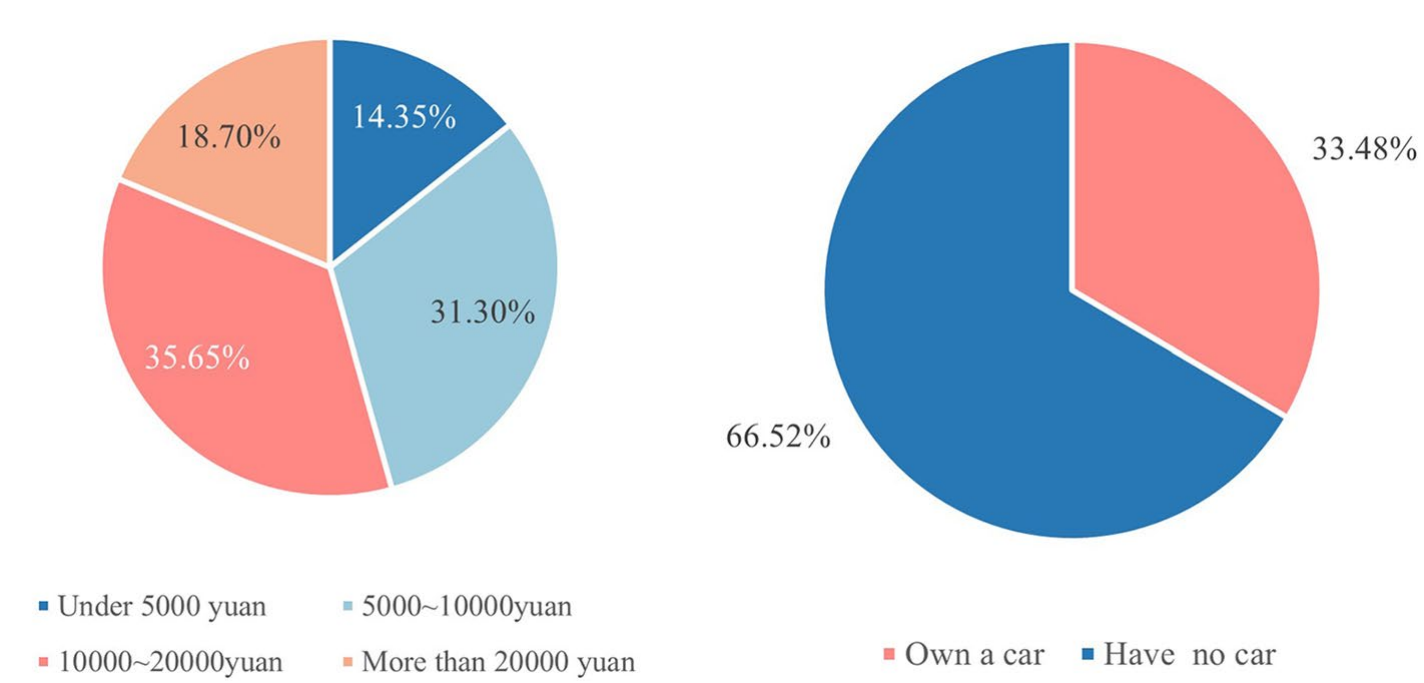
The income (left) and the ownership of private cars (right) of the respondents' households collected in the sample

As shown in Fig. 4, 66.52% of the respondents confessed that they or their families own a car; 33.48% of respondents do not own a car. Public data show that the number of cars per 100 households in Shanghai is around 70–80, and the socioeconomic results are consistent with the actual situation.

From the above basic personal characteristics, it is determined that the respondent group of this survey covers different gender, age, occupation, and income, which is relatively comprehensive.

#### Preliminary Analysis of RP Survey Results

The basic RP data are treated to analyze the situation of travelers on their most common suburban trips. From the perspective of travel purpose, 60.48% of the travel purpose is commuting, indicating that commuting is the most common purpose of urban-suburban travel.

From the perspective of travel choice, 65.32% of respondents choose the subway or suburban railway, 25.81% choose self-driving, carpooling, or taxi (online car-hailing), and 8.87% choose the ground bus.

From the perspective of travel time, 60.48% of the suburban travel time is more than 60 min, and 13.71% of the suburban travel time is more than 90 min. Regarding travel expenses, 51.61% of the respondents estimate their expenses to be less than 10 yuan. The vast majority (97.6%) of these low-cost-trip participants choose public transport, reflecting the low-cost character of public transport.

Regarding travel distance, 66.94% of the respondents estimate that their suburban travel distance is between 20 and 60 km, which is in line with the distance and time of suburban travel every day in Chinese megacities.

In terms of the way to get to the subway station or suburban railway station, 58.87% of the respondents reach the station by riding, short-distance shuttle, driving, or taking a taxi. The basic information about the data collected by the RP questionnaire is shown in Table 3.

**Table 3:** Basic information about the data collected by RP questionnaire

Question	Option	Proportion (%)
Travel purpose	Commuting	60.48
	Non-commuting	39.52
Travel mode	Rail transit	65.32
	Car	25.81
	Ground bus	8.87
One-way travel time	In 60 min	39.52
	60~90 min	46.78
	90~120 min	12.90
	Over 120 min	0.80
Estimated one-way travel cost	0~10 Yuan	51.61
	10~30 Yuan	33.06
	30~50 Yuan	8.06
	Over 50 Yuan	7.26
Estimated one-way travel distance	0~20 km	33.06
	20~30 km	36.29
	30~40 km	20.16
	40~60 km	9.68
	Over 60 km	0.80
How to get to a public transport station	Walk	41.13
	Cycling	26.61
	Driving or taxi	17.74
	Shuttle bus	14.52

#### Crossover Analysis

**Fig. 5:**
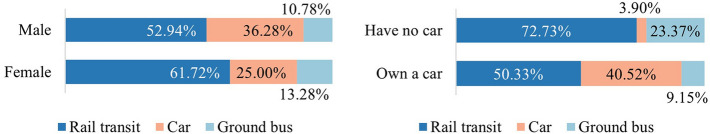
Crossover analysis of gender (or “CarOwn”) and travel mode choice

**Fig. 6:**
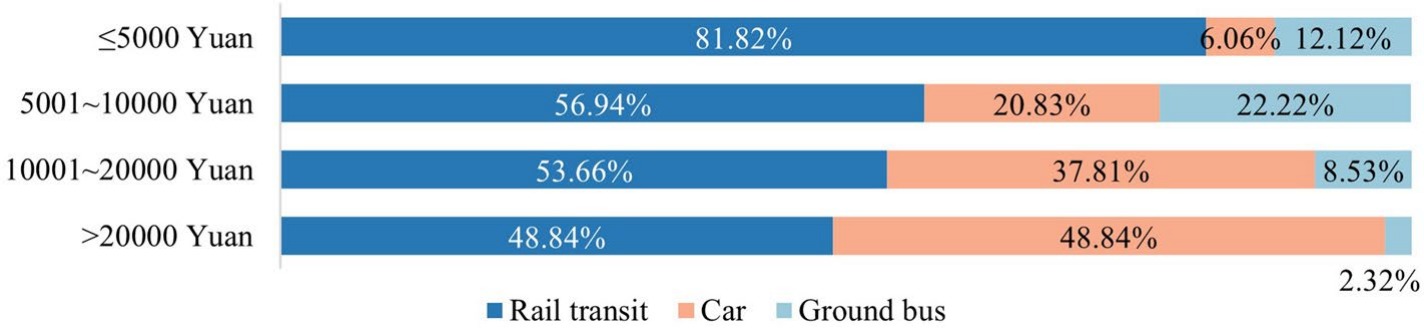
Crossover analysis of “Income” and travel mode choice

**Fig. 7:**
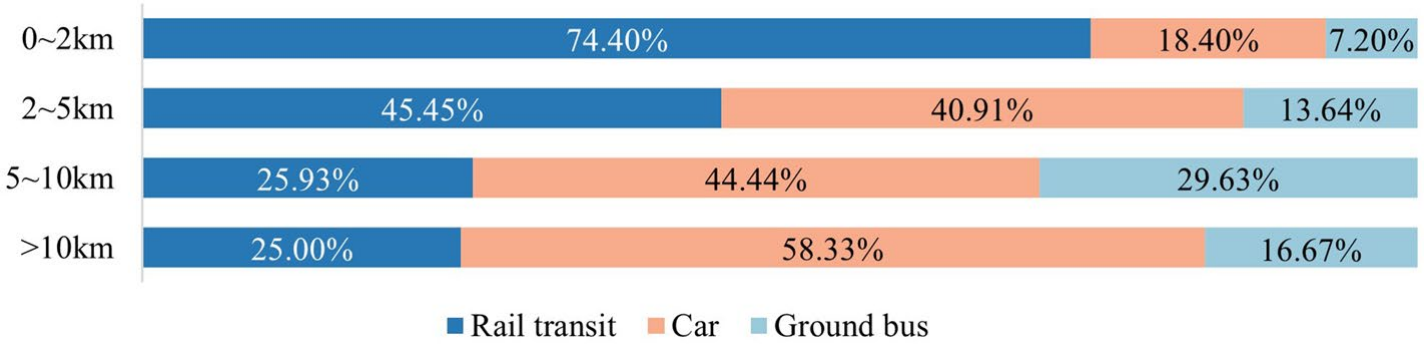
Crossover analysis of *D*_RS_ and travel mode choice

In the crossover analysis, we study the relationship between travel mode choice and certain attributes, which are shown in Figs. 5, 6 and 7. This analysis helped in the subsequent modeling.

It is observed that the probability of men choosing self-driving or taxis is higher than women, and women are more likely to choose public transportation than men. This situation may be due to the larger group of male drivers. The respondents with a car are significantly more likely to choose to drive themselves than those without a car.

Moreover, the higher the income of the respondents, the greater the proportion of not choosing public transport. In the income bracket of more than 20,000 yuan, about half of the respondents do not choose public transport for suburban travel. For the general respondents, the farther the rail transit station is from the destination or residence, the greater the proportion of people who choose car travel, and the rate of taking the ground bus also increases to a certain extent.

In the analysis of the cross-section of distance and travel mode, the pattern of “the further the distance from the metro or rail station, the less likely you are to choose rail travel” is basically present. In general, the further the rail station is from the destination or residence, the greater the proportion of trips made by car, and the rate of ground transportation increases to a certain extent.

### Modeling of Discrete Choice Model

This study uses a variety of discrete choice models, and finally uses the base MNL model with good modeling effect, the willingness-to-pay MNL model, the perceived-time MNL model, and the NL model. The independent variables in the models are the various factors mentioned above, while the dependent variables are the utility values that affect the probability of travel mode choice.

The results of the model parameter calibration are presented in Tables 4, 5, and 6. If the parameter estimate for this factor is blank, it means that there is no involvement of this factor in this model.

**Table 4:** Parameter estimation of the basic MNL model (Models 1 and 2)

Attribute	Options	Model 1: RP data modeling		Model 2: SP data modeling	
		Estimate	*T* value	Estimate	*T* value
Constant term	Rail transit	6.313732***	5.172001		
	Car	–	–		
	Ground bus	1.284963	0.958498		
Shared bikes available (AV_Bicycle)	Rail transit			0.329724***	3.697788
	Ground bus			0.556744***	5.827032
Shuttle buses available (AV_Shuttle)	Rail transit			0.218036**	2.459961
	Ground bus			0.508771***	5.384036
*P*+*R* available (AV_Parking)	Rail transit			−0.04505***	−4.11013
	Ground bus			0.024764***	−3.44516
Cost				−0.00539***	−4.06349
Travel time				−0.01503***	−22.1173
Possible delay	Rail transit			0.255149***	7.976195
	Car			−0.00107	−0.24284
	Ground bus			−0.01342***	−3.22719
Gender	Rail transit	0.907423*	1.769628	−0.21723***	−3.70796
	Ground bus	2.192218**	2.303239		
Income	Rail transit	−0.8671**	−2.71785	0.255442***	7.664403
	Ground bus	−1.11941**	−2.32927		
Crowded degree	Rail transit			−0.04653***	−4.84526
	Ground bus			−0.03365***	−3.3696
Distance (*D*_RS_+*D*_DS_)	Rail transit	−0.78156***	−4.44339	−0.0059	−0.41717
	Ground bus			0.732637***	6.823623
Car–own (for car option)		1.108377*	1.672457	0.952579***	14.33271
$L(\widehat{\theta })$		−75.14		−6235.58	
$L(0)$		−130.73		−6828.97	
$L(C)$		−101.17		−6738.35	
Degrees of freedom		8		18	
${\rho }^{2}$		0.4253		0.0869	
${\overline{\rho }}^{2}$		0.3641		0.0841	

– indicates that the influence is not significant, *** indicates that the probability of significantly influencing the choice of mode is above 99%, ** indicates that the probability of significantly influencing the choice of mode is above 95%, * indicates that the probability of significantly influencing the choice of mode is above 90%. The same applies below.

**Table 5:** Parameter estimation of the willingness-to-pay MNL model (Model 3) and perceived-time MNL model (Model 4)

Attribute	Options	Model 3: Willingness-to-pay MNL		Model 4: Perceived-time MNL	
		Estimate	*T* value	Estimate	*T* value
Perceived time (for perceived-time MNL)				−0.00819***	−5.83618
Shared bikes available (AV_Bicycle)	Rail transit	0.348192***	3.879571	−6.98042***	−3.07426
	Ground bus	0.558165***	5.82902	−42.2342***	−4.05665
Shuttle buses available (AV_Shuttle)	Rail transit	0.235085***	2.63556	−5.76653***	−2.65911
	Ground bus	0.509806***	5.38297	−41.4365***	−4.02736
*P*+*R* available (AV_Parking)	Rail transit	−0.03333***	−3.94422	6.091915***	2.832883
	Ground bus	0.032799***	−3.4713	−23.65791***	2.75457
Cost		−0.00642***	−4.76591	−0.0066***	−4.94546
Travel time	Rail transit	2.796836***	4.679081		
	Car	1.511842***	3.687513		
	Ground bus	2.16998***	4.59389		
Possible delay	Rail transit	−68.1341***	−4.05864	−51.7579***	−4.33964
	Car	−0.0152	−0.02208	−0.02837	−0.05305
	Ground bus	2.073313***	2.602269	1.820785***	3.04754
Gender		33.49051***	2.963006	−0.21254***	−3.64316
Income		−39.7408***	−4.09529	0.255057***	7.695162
Crowded degree	Rail transit	6.622462***	3.132012	1.261394***	3.130479
	Ground bus	4.638946***	2.602161	0.696598***	2.343788
Distance (*D*_RS_+*D*_DS_)	Rail transit	0.583502	0.261867	1.065058	0.541214
	Ground bus	−177.617***	−3.84911	−136.489***	−4.07994
Car-own (for car option)		−123.286***	−4.40033	0.791534***	11.21091
$L(\widehat{\theta })$		−6235.58		−6235.58	
$L(0)$		−6828.97		−6828.97	
$L(C)$		−6738.35		−6738.35	
Degrees of freedom		20		18	
${\rho }^{2}$		0.0879		0.0869	
${\overline{\rho }}^{2}$		0.085		0.0841

**Table 6:** Parameter estimation of the NL model (Model 5 and Model 6)

Attribute	Options	Model 5: NL model + RP data		Model 6: NL model + SP data	
		Estimate	*T* value	Estimate	*T* value
Constant term	Rail transit	−2.53332	–		
	Car	−1.25217	–		
	Ground bus	3.785483	–		
Shared bikes available (AV_Bicycle)	Rail transit			0.3424***	3.977933
	Ground bus			0.512311***	5.603991
Shuttle buses available (AV_Shuttle)	Rail transit			0.237243***	2.77546
	Ground bus			0.456697***	5.025923
*P*+*R* available (AV_Parking)	Rail transit			−0.03539***	−4.38551
	Ground bus			0.134384***	−3.94306
Cost				−0.0063***	−4.75914
Travel time				−0.0126***	−11.193
Possible delay	Rail transit			0.348442***	10.47697
	Car			0.001223	0.276004
	Ground bus			−0.0092***	−2.61489
Gender	Rail transit	0.907489*	1.769058	−0.21125***	−3.62157
	Ground bus	2.195168**	2.146022		
Income	Rail transit	−0.86754**	−2.6891	0.252708***	7.616108
	Ground bus	−1.12086**	−2.18604		
Crowded degree	Rail transit			−0.03774***	−4.43267
	Ground bus			−0.02761***	−3.37983
Distance (*D*_RS_+*D*_DS_)	Rail transit	−0.7827***	−3.4783	−0.0076	−0.65837
	Ground bus			1.044805***	9.503688
Car-own (for car option)		1.108415*	1.67216	0.77982***	11.04306
NL model-specific parameters		1.003157**	2.548633	0.753528***	9.451684
$L(\widehat{\theta })$		−75.14		−6381.73	
$L(0)$		−130.73		−6959.71	
$L(C)$		−101.17		−6857.07	
Degrees of freedom		10		19	
${\rho }^{2}$		0.4253		0.083	
${\overline{\rho }}^{2}$		0.3488		0.0803	

#### The MNL Model

1. Model 1: RP data modeling

In modeling RP data, we consider that the utility functions of the three ways have constant terms. On the one hand, whether to own a car will affect the probability of choosing a car for travel; on the other hand, people with higher income levels are more inclined to travel by car. At the same time, because statistics show that male drivers account for the majority, gender differences may also be related to the travel mode choice.

The discrete choice model program package Apollo of *R* language is used to estimate parameters and test parameters based on RP survey data and the above model, and the parameters with insignificant influence are eliminated. Finally, the parameter calibration results are obtained, as shown in Table 4.

According to the parameter estimation results, the distance to the station is the most significant variable. The coefficient is negative, indicating that the farther the distance between the destination and the rail transit station is, the fewer travelers are willing to choose rail transit travel. The income level also has a significant impact on the travel mode, which is negative for rail transit and ground bus, and the absolute value of the ground bus coefficient is larger, indicating that when other conditions are unchanged, the higher the income, the less willingness to choose public transportation, especially the ground bus.

The coefficient of owning a car on traveling by car is positive, and the absolute value is large, which reflects that “car owners” are significantly more willing to choose car travel. In terms of gender, according to the preliminary analysis of the data in the previous chapter, it is believed that women are more likely to choose public transportation than men.

(2) Model 2: SP data modeling

Based on the modeling of RP data, SP data is modeled. The coefficients of “car ownership,” “gender,” and “income” only exist in the utility function of the car, and the coefficients of *D*_RS_ and *D*_DS_ only exist in the utility function of the rail transit and bus, and the coefficients of “whether to use a certain connection mode” and “crowding degree” only exist in the utility function of the rail transit and public transit. The final parameter calibration results are obtained as shown on the right side of Table 4, next to the results of Model 1.

Overall, most of the travel attribute factors have a significant impact on the choice of travel mode, and the significance of the travel time is of the highest and negative value, reflecting that the length of the travel time is still one of the most important travel attributes for travelers to choose travel modes.

Income and car-own, two factors with high significance in the RP model, are also very significant in the SP model. A positive value indicates that people with higher incomes and cars are more willing to choose cars for travel.

In terms of the congestion degree, the test value is significant, while the absolute value of the parameter is not large, indicating that the effect of the congestion degree of public transportation is low, but this could also be due to the level of crowding is not communicated well to the respondents.

The impact of possible delay on mode choice is more intense in public transportation, and for cars, the estimated value is small and insignificant, which can be understood as residents who choose to travel by car having a psychological expectation of road congestion and possible delay.

The test values of all parameters are relatively significant in terms of the three connection methods. The most favored connection method of respondents is the shared bicycle, followed by shuttle car, and finally “*P*+*R*.”

#### The Willingness-to-pay MNL Model and Perceived-time MNL Model

Based on the SP-based MNL model in the previous section, the attributes such as travel time, congestion degree, and possible delay with their respective parameters, plus the travel cost and multiply the sum by the cost coefficient, are in the willingness-to-pay MNL (WTP-MNL) model (Model 3). The calibration results are shown in Table 5.

The parameter estimation of the WTP-MNL model is more significant than the basic MNL model overall, and the characteristics of the factors are similar to the basic MNL model.

Based on the SP-based MNL model in the previous section, the *D*_RS_ and *D*_DS_ coefficient is modified by the product of the connection mode, considering that the connection tool will affect the distance between arrival and departure stations. On the other hand, the sensing time of the crowding degree is related to the traveling time, so the product of the two is multiplied by the crowding coefficient alone. Finally, these “sensing time terms” are added to the traveling time to obtain the total sensing time and then multiplied by the coefficient of the sensing time, which is the sensing time-MNL model (Model 4). The obtained utility function and parameter calibration results are shown in Table 5, next to the results of Model 3.

#### NL Model

**Fig. 8:**
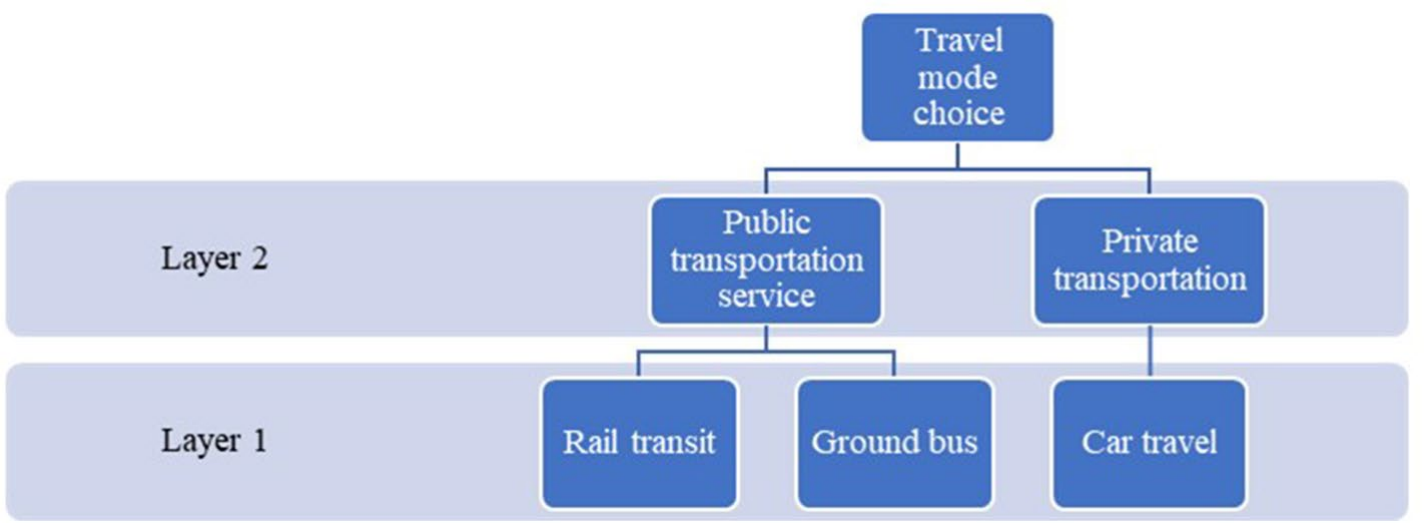
Schematic of the NL model

Based on the basic MNL model of RP and SP data, considering that rail transit and ground public transportation are similar in timeliness, economy, comfort, and other aspects, a two-layer tree structure is constructed, and “rail transit” and “ground bus” are classified as “public transportation service,” and the selection tree is as following Fig. 8.

Then we modify Model 1 (RP data) and Model 2 (SP data) into Model 5 and Model 6, respectively, using the selection tree above. The results of parameter calibration in the Apollo package are shown in Table 6.

In the parameter calibration of the NL model based on RP results, its overall significance is not as good as that of the basic MNL model, which may be because RP data is based on actual travel behavior. There are still factors not involved in the survey in the actual travel behavior to participate in the choice of travel modes, and there is no strong similarity within public transportation.

In the parameter estimation based on SP data, the overall significance is stronger than that of the basic MNL model, and the characteristics and disadvantages of the factor are similar to that of the basic MNL model.

### Modeling of Machine Learning Methods

This study selects two common machine learning algorithms for classification problems. Based on the same set of SP survey data (15540 data is divided into 75% training set and 25% test set), the two algorithms are used to build a trip choice model and test its accuracy. The results are compared with those under the discrete choice model.

#### Support Vector Machine

Support vector machine (SVM) is a supervised learning algorithm for data classification. Its advantages are that it can learn based on smaller samples, emphasize key vectors, is not too sensitive to outliers, and has strong generalization ability.

In this study, the SP training set is modeled using the LIBSVM program package based on R Language, which is commonly used in data science research. After parameter adjustment, when the function type of nonlinear transformation is the Gaussian kernel function, and the penalty function is set to 3 (gamma=0.7), the prediction accuracy of the test set is the highest, reaching 63.84%.

#### Random Forests

In this study, *randomForest*, a program package based on *R* language, is used to model the training set. When the decision tree size is 2500, the prediction accuracy of the training set is the highest, reaching 63.11%, and the overall accuracy of the test set is 62.68%.

**Fig. 9:**
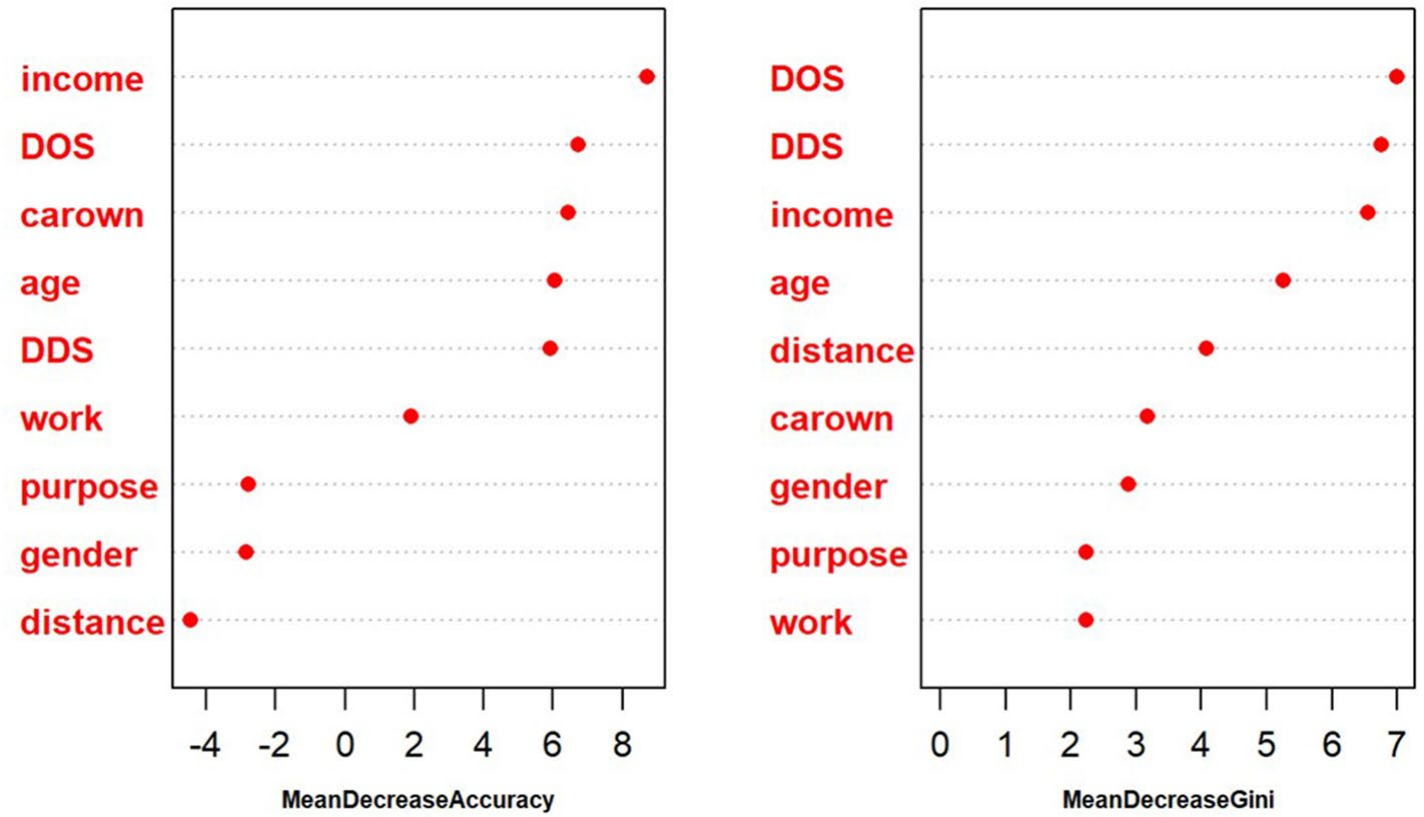
Importance ranking of factors in RP models by random forest

**Fig. 10:**
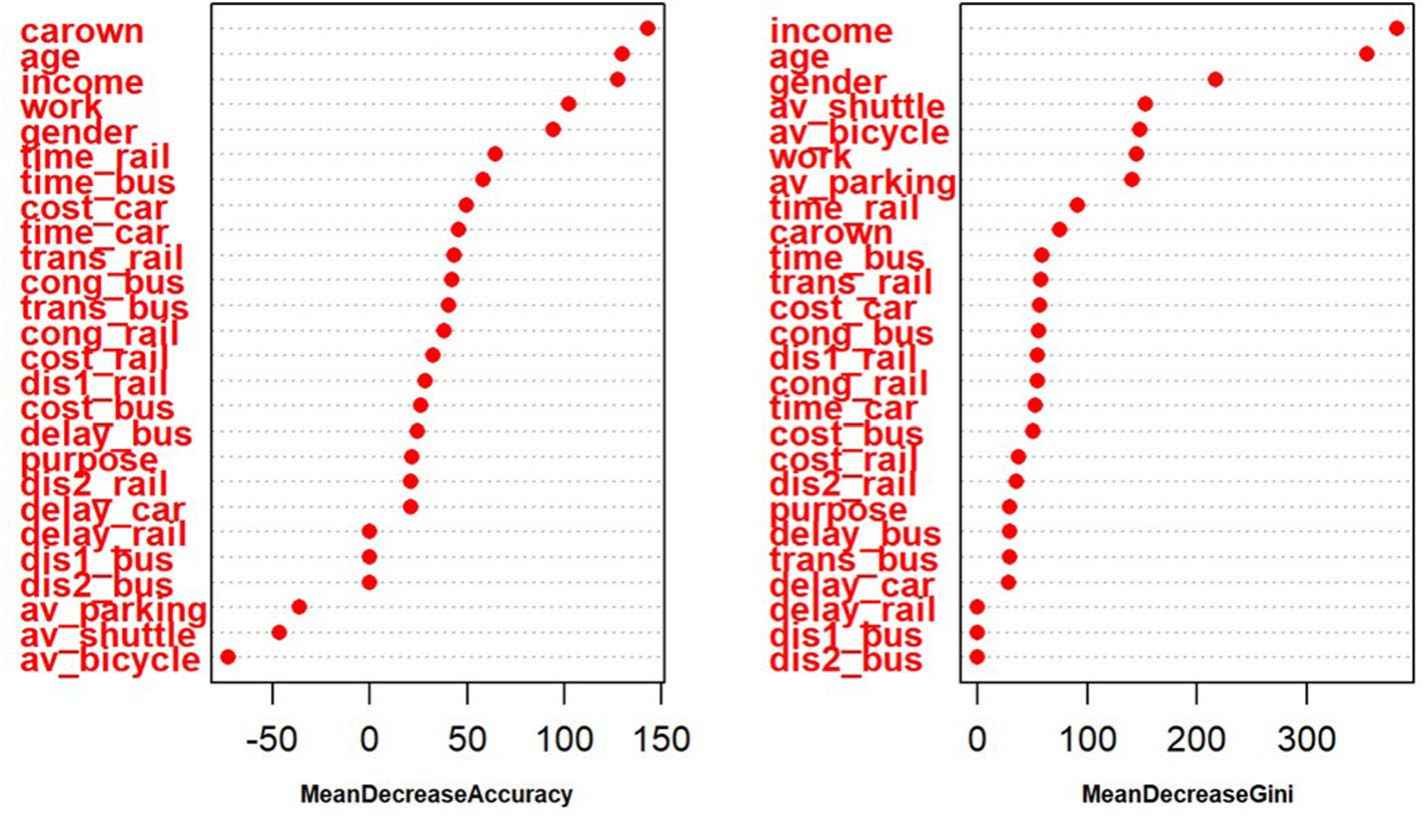
Importance ranking of factors in SP models by random forest

In addition, the importance order of each factor in SP and RP data is printed, as shown in the following Figs. 9 and 10. Each figure has two diagrams. Mean decrease accuracy represents the degree of reduction in the prediction accuracy of the random forest when this option is randomly selected. Mean decrease Gini computes the effect of each variable on the heterogeneity of observations at each node of the classification tree, thus comparing the importance of variables. The higher the value, the more important the variable is.

According to the factor importance ranking of RP data (shown in Fig. 9), it is found that work, travel purpose, gender, and total travel distance are low in the two diagrams and *D*_RS_ and *D*_DS_ (DOS and DDS in Fig. 9), income, and age are high. The variable “whether to own a car” ranks third in the accuracy diagram. The low ranking in the Gini diagram is probably due to its binary nature.

In ranking factor importance based on SP data (shown in Fig. 10), factors such as income, age, gender, job, and whether to own a car rank high, and the ranking of travel time is also relatively high. The rank of crowding degree is in the middle. Possible delays and *D*_RS_ and *D*_DS_ (dis1 and dis2 in Fig. 10) are low on the list.

Additionally, in DCM models the parameters of some factors have to be uniform and cannot be differentiated by travel modes in order to ensure significance (for example, factors such as cost and time have to be uniformly parameterized). However, in a random forest model, researchers can obtain more detailed results. For example, in the ranking of mean decrease accuracy and mean decrease Gini, travel time by rail is more important than time by bus or car, and people are most concerned about the crowdedness and possible delays of buses and cars. In terms of the cost factor, it is the cost of travel by private car that has the greatest impact.

### Major Findings

The following main findings can be obtained by combining the parameter estimation and factor importance ranking of the above models:

1. **Time has more influence on travel choice and may be greater than the impact of cost.** In all models, the time parameter continues to have strong significance. In the random forest model, the importance of the time parameter on the road is also relatively high.
2. **Suburban railways show advantages in the time–cost ratio.** The timeliness of the rail transit network, including suburban railways, is enhanced (30–60 min can be saved), and the travel cost is not significantly increased (10 yuan for Jinshan Line and assume 10 to 15 for other suburban lines in Section 4) compared to the metro ticket. Compared with cars, the advantage of the low price of public transit is retained.
3. **Among the three connection modes, the proportion of travelers influenced by them to use public transit: Shared bicycle>short distance shuttle > *P*+*R*.** Considering the dense bus network stations, many connection modes may be more valuable in the travel chain dominated by rail transit.
4. **The delay coefficient of car travel is not obvious in the parameter estimation.** The absolute value is small, which may be because the passengers who choose car travel have a psychological expectation for the possible delay in highway traffic.
5. **Travelers who own cars are stickier to car travel, which is reflected in the strong significance of the Car-own coefficient.** Moreover, because of their high income (reflected by the income parameter), they are less sensitive to direct travel costs than public transport travelers. On the contrary, travelers who do not choose cars or have not yet purchased a car are also stuck to public transport, which means that they generally do not change their public transport travel rules.

## Prediction of the Effect of Transportation Demand Management Schemes

According to the results in previous sections, considering each model's advantages and disadvantages, the basic MNL-SP model, NL-SP model, and support vector machine model with better overall performance are selected to predict the change in travel mode choice under 19 different policy schemes. Predictions of the changes in travel mode choices are made under these assumptions. The 19 schemes and predicted results are shown in Table 7.

**Table 7:** The change value of the proportion of travel choice under different traffic demand management schemes

The change value of the proportion of travel choice under different schemes (%)										
Traffic demand management scheme		MNL			NL			SVM		
		Rail	Car	Bus	Rail	Car	Bus	Rail	Car	Bus
Rail transit timeliness increased	1. Rail travel time <90 min	8.5	−2	−6.5	9.7	−1.9	−7.9	6.1	−3.2	−2.9
	2. Rail travel time < 60 min	24.1	−9.7	−14.5	24.5	−7.9	−16.6	9.1	−4.6	−4.5
	3. Rail travel time −15%	10.4	−4.8	−5.6	8.4	−3.4	−5			
	4. Rail travel time −30%	22.5	−10.1	−12.4	22.5	−7.7	−14.8			
Rail transit attributes improved	5. Rail crowded degree <6	4.4	−1.8	−2.7	4.4	−1	−3.4	7	−3.6	−3.4
	6. Distance from station to residence/destination	0.3	−0.3	0	0.7	−0.7	0	7.4	−4.3	−3.1
Restricting car travel	7. Car cost +50%	10.6	−4.6	−6	3.3	−5.1	1.8			
	8. Car cost +100%	14.8	−14.2	−0.6	5.1	−8.5	3.4			
	9. Private cars banned and taxis only	17.9	−15.6	−2.3	3.2	−5.9	2.7	10.1	−5.5	−4.6
Connecting traffic modes	10. Shuttle bus + shared bicycle	26	−10.8	−15.3	32.3	−11.7	−20.6	3.7	−4	0.4
	11. Shuttle bus + “*P*+*R*”	−3.7	9.2	−5.4	−4.9	5.9	−1	2.7	−1.3	−1.4
	12. Shared bicycle + “*P*+*R*”	0.1	5.7	−5.8	2.3	1	−3.4	3.6	−1.7	−1.9
	13. Shared bicycle + “*P*+*R*” + shuttle bus	14	−2.9	−11.2	17.3	−6.1	−11.2	6.2	−4.4	−1.8
Implement multiple measures at the same time	14. Rail travel time −15% and car cost +50%	19.7	−11.1	−8.6	18.7	−8	−10.7			
	15. Rail travel time −15% and car cost +100%	23.7	−16.8	−6.9	21.3	−11.8	−9.6			
	16. Rail travel time −15% and taxis only	26.5	−19.3	−7.3	20.5	−10.8	−9.6			
	17. Rail travel time −30% and car cost +50%	29.4	−14.9	−14.5	28.4	−11.4	−17			
	18. Rail travel time −30% and car cost +100%	33.9	−20	−13.9	30.5	−14	−16.5			
	19. Rail travel time −30% and taxis only	36.8	−22.6	−14.2	30.3	−13.9	−16.3			

In Section 6.1, we will explain why these schemes are proposed and show the predictions of these schemes. Our suggestions are derived from the research in Sections 4 and 5 and are presented in 6.2.

### Schemes and Effect Predictions

#### Improve Timeliness of Rail Transit

The timeliness of the travel mode is still a key factor for travelers. This study considers the increase in timeliness from two perspectives. Schemes 1 and 2 are designed to assume the disappearance of extremely long-time trips, while Schemes 3 and 4 are aimed at a general reduction in travel time.

The results show that the effect of Scheme 1 is like that of Scheme 3, and the effect of Scheme 2 is like that of Scheme 4. However, the effect of Schemes 2 and 4 is much more significant than Schemes 1 and 3, and are among the most effective among 19 schemes. The results suggest that Shanghai should continue to promote the construction of municipal railways and subways to improve the timeliness of rail transit.

#### Improve the Portability of Rail Transit

Apart from the cost, time, and connection method, the congestion degree and the distance between the station and the residence or destination are the most significant travel mode factors. Therefore, Scheme 5 is to improve comfort (reduce carriage congestion), and Scheme 6 is to improve convenience (to shorten the distance between rail transit stations and travelers). The prediction results show that these two schemes have a relatively weak influence.

#### Improve the Accessibility of Rail Transit

In the SP survey, for each scenario, each respondent was provided with three access modes to and from public transport stations: shuttle bus, shared bicycle, and *P*+*R*. We examine the impact of providing multiple connection methods simultaneously by Schemes 10 to 13.

According to the table of prediction results, when there are both shuttle cars and shared bicycles (Scheme 10), the impact is the most significant, and schemes 11 and 12 have a small impact. The *P*+*R* mode is more expensive and laborious than other connection methods in construction and management, and the development of the *P*+*R* mode in China is not mature enough. Choosing the appropriate placement of shared bicycles and the reasonable arrangement of connecting buses is better.

#### Restrict Car Travel

Although car travelers may be less sensitive to the increase in travel costs, increasing the cost of car travel is a common and practical travel demand management measure. The government usually uses congestion pricing (Schemes 7 and 8), driving restrictions based on license plates (Scheme 9), and other measures to curb the number of private cars.

Schemes 7 and 8 assume that the car travel cost increases by 50% and 100%, respectively. The results show that the impact is significant.

Scheme 9 assumes that people can no longer travel by themselves but choose public transit or taxis (no carpooling), and the cost of car options is increased to the taxi price level (80 yuan). The results show that scheme 9 has a relatively significant impact on the basic MNL model.

#### Using Multiple Methods

According to the results of schemes 14 to 19, it is found that when time and cost measures are used together, the overall impact is more significant. Multiple measures can be considered in specifying the competitiveness of suburban public transport services, which may be better than implementing one measure alone.

### Suggestions to enhance competitiveness of public transit

1. **Improving the timeliness of the public transport system through efforts to develop suburban railways.** The predictions in Schemes 1 to 4 show significant passenger demand for more excellent timeliness in the public transport system. If travel times can be reduced by 30%, or extra-long trips over 60 min can be eliminated, the rail share could be significantly increased.
  The Shanghai Metro generally travels at speeds below 40 km/h, with most trains stopping at every station and shorter station spacing (generally 1–5 km). In this case, consideration should be given to improving the timeliness of the rail network through the development of a faster (fastest travel speed of around 100 km/h) suburban railways with fewer stations and greater station spacing (generally 3–10 km).

(2) **It is desirable that suburban rail pricing remain largely consistent with metro pricing rates.** In the SP experiment we found that if other suburban lines were kept at the same price level of no more than 15 yuan as the Jinshan Railway (10 yuan), they would not only have the advantage of timeliness, but their price advantage would be substantially retained. If it were to work with the metro to offer various combined fares, it might attract more passengers and get them into the habit of traveling by public transport.
  Because of the high investment and low pricing, it is difficult for municipal rail operators to profit in the short term. Therefore, subsidies could be provided to the operators to ensure the long-term operation of the transport services, or allow them to profit from land sales and real estate development.

(3) **Scientific placement of shared bikes near stations and running shuttle buses.** Schemes 10 to 14 demonstrate that shared bikes and shuttle buses are the more significant connection methods in terms of impact. Bicycle sharing has the most significant impact overall, with the added benefit that its investment often comes from private companies and is less costly for the government.
  Shuttle buses are preferred by those who cannot cycle or are in bad weather. Priority can be given to smaller, more frequent, demand-responsive forms of public transport to reduce waiting time for transfers. In order to achieve the goal of linking the various modes of transport, consideration could be given to integrating the operators of the public transport system, to provide a more complete transport service.

(4) **Using methods such as license restrictions to increase the cost of car travels.** Schemes 8 and 9 contribute more significantly to the increase in rail sharing. The prediction results show that for every 10% increase in the cost of car travel, the rail share rate is likely to increase by 1–2%. This reflects the continued sensitivity of travelers to the cost of traveling by car. Considering that license restrictions have been implemented in some Chinese cities, policymakers can continue to consider this policy while looking at other measures, such as reducing subsidies for car purchases and increasing parking fees.
(5) **The combination of multiple types of measures is more effective than a single measure.** The results of schemes 14 to 19 can prove this.

## Conclusions

This paper focuses on the passengers' travel choice behavior in urban-suburban passenger transport corridors. Based on the collected RP and SP data, we apply the discrete choice models and machine learning methods to construct the models, which are further compared and comprehensively analyzed. At the same time, this research focuses on the impact level of each factor on the selection decision considering a newly built urban railway or providing connection services to the suburban public transit stations.

The following findings are obtained.

1. It is found that time and cost play a vital role in travel choice, and the low price of suburban railways (10–15 yuan) compared with car travel still retains the advantage of the low price of public transport, which is attractive to many travelers.
2. Rail transit passengers in Shanghai prefer shared bicycles and shuttle buses on the “last mile” trips, but the *P*+*R* transfer method is not as attractive.
3. People who do not use public transport are more insensitive to the price and remain more tolerant of predictable congestion and delays. Strong charging policies such as congestion charges, traffic bans, and restrictions on car purchase qualifications may discourage car travel.

Based on the above findings, we make the following suggestions.

1. Shanghai should continue to promote the planning and construction of the suburban railway and maintain the one-way price level at 10–15 yuan.
2. Scientific placement of shared bikes or logical operation of feeder buses near stations can improve the accessibility of rail transit.
3. Considering the construction and operation costs, the government needs to provide certain subsidies to stabilize prices.
4. Use methods such as license restrictions to increase the cost of cars because participants are sensitive to the cost of car travel.
5. Implementing multiple measures in traffic demand management is also suggested, which has a significantly better effect than a single measure.

This study uses discrete choice models and machine learning algorithms to construct a travel mode choice model of suburban passenger transport corridors. In Section 5, the results of the two methods support each other, highlighting the most significant and influential factors, such as cost and time. In Section 6, based on the findings of the previous section and considering the social and transport development of Shanghai, 19 schemes are proposed. Conclusions and suggestions are obtained by comparing the prediction results of the two types of models.

Additionally, the research and prediction methods that combine actual conditions with different algorithms can provide application data support in speed designs, station spacings, and connecting traffic modes when designing or operating public transport services.

However, the collected RP and SP data in this study are not fused because of the difference in data sizes. Further studies may consider integrating the two approaches used in this paper, in which the role, value, and effect of travel mode choice need to be explored. This paper does not further integrate the two approaches in use. Further studies can continue exploring the value of combining new methods (such as machine learning) with the DCM in travel mode choice, studying the effect it can bring when used with the traditional discrete choice models.

## Supplementary Information

Below is the link to the electronic supplementary material.

Supplementary file 1 (DOCX 20 kb)

Supplementary file 2 (DOCX 24 kb)
