# Supplementary material for: Understanding Influencing Factors of Travel Mode Choice in Urban-Suburban Travel: A Case Study in Shanghai
Source: Urban Rail Transit. 2023 Mar 26:1–20. Online ahead of print. doi: 10.1007/s40864-023-00190-5 (PMC10040232; doi:10.1007/s40864-023-00190-5)
Supplement: Supplementary file 1 — (DOCX 20 kb) [file 40864_2023_190_MOESM1_ESM.docx]

**All 24 scenarios in the SP survey part of the questionnaire**

| **Scenario** | Travel purpose | **Rail transit** | | | | | | | **Car** | | | **Ground bus** | | | | | |
| --- | --- | --- | --- | --- | --- | --- | --- | --- | --- | --- | --- | --- | --- | --- | --- | --- | --- |
|  |  | Travel time  /min | Cost  /Yuan | D_OS_  /km | D_SD_  /km | Crowded degree  / people per m^2^ | Possible delay  /min | Number of transfers | Travel time  /Min | Cost  /Yuan | Possible delay  /min | Travel time  /min | Cost  /Yuan | D_OS_  /km | D_SD_  /km | Possible delay  /min | Crowded degree  / people per m^2^ |
| **1** | 1 | 120 | 5 | 5 | 3 | 10 | 5 | 1 | 40 | 30 | 30 | 120 | 5 | 1 | 0.5 | 30 | 10 |
| **2** | 1 | 60 | 15 | 5 | 3 | 6 | 5 | 0 | 40 | 50 | 15 | 90 | 2 | 1 | 0.5 | 15 | 3 |
| **3** | 0 | 90 | 5 | 1 | 1 | 10 | 5 | 1 | 60 | 50 | 30 | 60 | 2 | 1 | 0.5 | 30 | 10 |
| **4** | 0 | 40 | 15 | 1 | 3 | 6 | 5 | 1 | 40 | 80 | 15 | 40 | 10 | 1 | 0.5 | 15 | 10 |
| **5** | 0 | 60 | 10 | 1 | 3 | 10 | 5 | 0 | 40 | 30 | 15 | 60 | 5 | 1 | 0.5 | 30 | 3 |
| **6** | 0 | 120 | 10 | 5 | 1 | 3 | 5 | 2 | 40 | 50 | 15 | 40 | 10 | 1 | 0.5 | 30 | 3 |
| **7** | 0 | 90 | 10 | 5 | 3 | 3 | 5 | 1 | 40 | 80 | 30 | 90 | 10 | 1 | 0.5 | 30 | 6 |
| **8** | 1 | 120 | 10 | 1 | 3 | 3 | 5 | 0 | 60 | 30 | 15 | 90 | 10 | 1 | 0.5 | 15 | 3 |
| **9** | 1 | 40 | 10 | 3 | 1 | 6 | 5 | 1 | 40 | 80 | 30 | 90 | 2 | 1 | 0.5 | 30 | 3 |
| **10** | 0 | 120 | 5 | 1 | 1 | 6 | 5 | 2 | 40 | 30 | 15 | 90 | 2 | 1 | 0.5 | 30 | 6 |
| **11** | 0 | 60 | 15 | 3 | 1 | 10 | 5 | 1 | 60 | 80 | 15 | 90 | 10 | 1 | 0.5 | 30 | 10 |
| **12** | 1 | 40 | 10 | 1 | 3 | 10 | 5 | 1 | 90 | 50 | 15 | 90 | 10 | 1 | 0.5 | 30 | 6 |
| **13** | 0 | 60 | 5 | 3 | 1 | 3 | 5 | 2 | 40 | 50 | 15 | 120 | 5 | 1 | 0.5 | 15 | 10 |
| **14** | 0 | 60 | 5 | 5 | 1 | 6 | 5 | 1 | 90 | 30 | 30 | 90 | 10 | 1 | 0.5 | 30 | 3 |
| **15** | 1 | 90 | 5 | 1 | 1 | 6 | 5 | 2 | 60 | 80 | 15 | 120 | 5 | 1 | 0.5 | 30 | 3 |
| **16** | 0 | 40 | 15 | 3 | 3 | 3 | 5 | 1 | 90 | 30 | 15 | 90 | 5 | 1 | 0.5 | 30 | 3 |
| **17** | 0 | 120 | 10 | 3 | 3 | 6 | 5 | 2 | 60 | 50 | 30 | 60 | 5 | 1 | 0.5 | 30 | 6 |
| **18** | 1 | 90 | 10 | 3 | 3 | 6 | 5 | 0 | 90 | 30 | 15 | 120 | 2 | 1 | 0.5 | 30 | 10 |
| **19** | 1 | 60 | 15 | 1 | 1 | 3 | 5 | 2 | 90 | 80 | 30 | 120 | 2 | 1 | 0.5 | 30 | 6 |
| **20** | 1 | 90 | 5 | 3 | 1 | 10 | 5 | 1 | 40 | 30 | 15 | 40 | 10 | 1 | 0.5 | 15 | 6 |
| **21** | 0 | 40 | 10 | 5 | 1 | 10 | 5 | 1 | 60 | 30 | 15 | 120 | 2 | 1 | 0.5 | 15 | 6 |
| **22** | 1 | 60 | 5 | 5 | 3 | 3 | 5 | 2 | 60 | 80 | 15 | 60 | 2 | 1 | 0.5 | 30 | 10 |
| **23** | 1 | 120 | 10 | 5 | 1 | 10 | 5 | 1 | 90 | 80 | 15 | 90 | 5 | 1 | 0.5 | 15 | 10 |
| **24** | 0 | 120 | 5 | 3 | 3 | 10 | 5 | 0 | 90 | 80 | 15 | 120 | 2 | 1 | 0.5 | 15 | 3 |
