# Supplementary material for: Understanding Influencing Factors of Travel Mode Choice in Urban-Suburban Travel: A Case Study in Shanghai
Source: Urban Rail Transit. 2023 Mar 26:1–20. Online ahead of print. doi: 10.1007/s40864-023-00190-5 (PMC10040232; doi:10.1007/s40864-023-00190-5)
Supplement: Supplementary file 2 — (DOCX 24 kb) [file 40864_2023_190_MOESM2_ESM.docx]

**Questionnaire on urban-suburban travel modes of Shanghai residents**

Dear Sir/Madam,

We are researchers in transportation science. We are conducting a survey to better satisfy the demand for travel from the suburban arears to the main city in Shanghai. We sincerely invite you to spare some time out of your busy schedule to assist us in completing this questionnaire, which will help us to better analyze passenger travel preferences and will contribute to the integrated transport development of our city.

We are investigating travel behavior during a “regular epidemic prevention and control” period. Therefore, please ensure that your choices in this questionnaire are made without being affected by the lockdown due to the COVID-19 Epidemic.

All the information you provide will only be used for this research and the research process is confidential. Thanks for your support!

**I. Basic information**

1. What is your gender? [single-choice question]

2. What is your age? [Single choice]

Below 18, 18~30, 31~40, 41~50, 51~60, 61 or more

3. What is your current occupation? [Single choice]

civil servants, scientific, educational, medical workers, general employees of enterprises, business managers, self-employed owners or operators, farmers, students, soldiers, freelancers, retirees, and others.

4. What is your monthly family income per member? (Total of all income received from all sources, including pocket money) [Single choice]

Less than RMB5000, RMB5001-10000, RMB10001-20000, RMB20000 or more

5. Are you a resident of Shanghai? [Single choice]

I live permanently in Shanghai, I am visiting or staying in Shanghai for a short period of time

6. Which district in Shanghai do you currently live in? [Single choice]

7. Please estimate the distance from your place of residence to the nearest metro station? [Single choice]

0~2km, 2~5km, 5~10km , over 10km

**II. Please review your most frequent urban-suburban (from urban to suburban, or form suburban to urban) trips and answer the following questions.**

* Main city areas of Shanghai are defined in this questionnaire as: Huangpu, Jing'an, Xuhui, Changning, Yangpu, Hongkou, Putuo, and Pudong New Area (the part within the Central Ring Road). Suburbs are: Minhang, Baoshan, Jiading, Jinshan, Songjiang, Qingpu, Fengxian, Chongming and Pudong New Area (the part outside the Central Ring Road)

8. The district where your most common destination for suburban travel is located:

(If you live in an urban area, please select from 9 suburban options)

(If you live in the suburbs, please choose from 8 urban options) [Single choice]

9. Please estimate the distance of your destination from the nearest metro station? [Single choice]

0~2km, 2~5km, 5~10km , over 10km

10. What is the purpose of your trip? [Single choice]

Working and going to school, Leisure, Visiting friends and relatives, Shopping goods , Other

11. What is your typical mode of travel? [Multiple choice] *

Subway (metro),

Train (e.g. Jinshan Railway),

Self-driving (including carpooling),

Bus (including company bus),

Taxi (including online car-hailing)

12. What is your typical one-way travel time for this trip? [Single choice]

60 minutes or less , 60~90 minutes , 90~120 minutes , 120 minutes or more

13. What is the estimated cost of your one-way trip (including fuel cost and electricity)? [Single choice]

RMB0~10, RMB10~30, RMB30~50 , RMB50~100 , RMB100 or more

14. Please estimate the distance you will travel each way? [Single choice]

0~20km, 20~30km, 30~40km, 40~60km, 60km or more

15. How do you usually reach a public transport stop? [Single choice]

Walking, cycling, driving or taking a taxi, short shuttle bus

**Ⅲ. You will then answer 4 to 5 questions for each of the 6 sets of scenarios given.**

16. There are 3 travel modes, each with the following characteristics. which one would you choose in the following 5 different situations?

(This question is an example. Each respondent answered 6 random scenarios from questions 16 to 39, see "supplementary material_All scenarios in SP survey.docx" for all 24 scenarios)

Scenarios 1 (for example):

Situations:

| **Situations** | **Rail transit** | **Car** | **Ground bus** |
| --- | --- | --- | --- |
| 1. In general, what is your chosen travel mode? | **○** | **○** | **○** |
| 1. If a shuttle bus is opened between the place of residence and the station with more frequency and appropriate time, the one-way cost will increase by up to 3 yuan. What would be your travel mode choice in this situation? | **○** | **○** | **○** |
| 1. If the shared bicycles are placed near the residence and the station, the number is large, easy to park, and the travel cost is almost unchanged. What would be your travel mode choice in this situation? | **○** | **○** | **○** |
| 1. If there is a special public parking lot (P+R parking lot) next to the station with ample parking space available, and you can directly transfer to the subway after parking. The parking fee is 10 yuan/day. What would be your travel mode choice? | **○** | **○** | **○** |
| 1. If there is a railway from the city to the suburbs, when choosing "subway or train", the travel time can be shortened to 60 minutes, and the single trip cost becomes 15 yuan. What would be your travel mode choice? | **○** | **○** | **○** |
